# Fungal Apoptosis-Related Proteins

**DOI:** 10.3390/microorganisms12112289

**Published:** 2024-11-11

**Authors:** Longjie Li, Chunmei Du

**Affiliations:** Engineering Research Center of Agricultural Microbiology Technology, Ministry of Education, Heilongjiang Provincial Key Laboratory of Plant Genetic Engineering and Biological Fermentation Engineering for Cold Region, Key Laboratory of Microbiology, College of Heilongjiang Province, School of Life Sciences, Heilongjiang University, Harbin 150080, China; yanmoran1212@126.com

**Keywords:** apoptosis, programmed cell death, apoptosis-inducing factor, metacaspase, inhibitors of apoptosis proteins

## Abstract

Programmed cell death (PCD) plays a crucial role in the development and homeostasis maintenance of multicellular organisms. Apoptosis is a form of PCD that prevents pathological development by eliminating damaged or useless cells. Despite the complexity of fungal apoptosis mechanisms being similar to those of plants and metazoans, fungal apoptosis lacks the core regulatory elements of animal apoptosis. Apoptosis-like PCD in fungi can be triggered by a variety of internal and external factors, participating in biological processes such as growth, development, and stress response. Although the core regulatory elements are not fully understood, apoptosis-inducing factor and metacaspase have been found to be involved. This article summarizes various proteins closely related to fungal apoptosis, such as apoptosis-inducing factor, metacaspase, and inhibitors of apoptosis proteins, as well as their structures and functions. This research provides new strategies and ideas for the development of natural drugs targeting fungal apoptosis and the control of fungal diseases.

## 1. Introduction

Cell death can be divided into two main types. One type is accidental cell death (ACD), which is uncontrollable and caused by external physical, chemical, or mechanical stimuli. The other type is regulated cell death (RCD), which can be controlled through specific drugs or genetic interventions [1]. When this RCD occurs as part of a developmental program or to maintain the physiological balance of adult tissues, it is referred to as programmed cell death (PCD). PCD is crucial for the development of multicellular organisms, organ sculpting, and the maintenance of cellular homeostasis [2]. Apoptosis is a significant form of PCD, representing an effective self-destruction mechanism by which cells respond to critical apoptotic signals to eliminate senescent, infertile, or damaged cells. Inappropriate or erroneous regulation or inhibition of apoptotic capabilities may contribute to the aging process and the development of certain pathologies. The apoptotic mechanisms in metazoans are relatively well understood, but the occurrence of apoptosis in fungi has been a subject of debate [3]. The first observation of cell death phenomena with apoptotic characteristics in yeast was reported in 1997 [4]. Subsequently, it was discovered that apoptosis-like PCD may exist in all unicellular and multicellular fungi and is regulated through highly specialized and sophisticated mechanisms [5,6,7,8,9,10]. The complexity of fungal apoptotic mechanisms and their background pathways bear some comparability to those of plants and metazoans [11]. Yet, the core regulatory components and pathways of animal apoptosis are absent in fungi [12], indicating that the underlying mechanisms of fungal apoptosis differ from those of animals [3,13,14]. Therefore, fungal apoptosis is often referred to as apoptosis-like cell death. Apoptosis-like cell death in fungi can be triggered by external stimuli, such as viral infections, oxidative stress, nutrient competition, and drug induction, or by internal stimuli, such as aging, mutation, and non-compatible signals [11,13,15]. It is one of the main strategies for fungi to resist environmental stress and maintain homeostasis. It is involved in a variety of important biological processes, including growth, development, stress response, aging, and host-pathogen interactions [16,17]. The clearance of apoptotic cells contributes to the formation of a healthier and more environmentally adapted population [18,19].

Although the role and regulatory mechanisms of apoptosis-like PCD in many biological processes in fungi have not been fully explored, it appears that the instability of the plasma membrane is a common mechanism [11]. In most cases, successful apoptosis is triggered by the mitochondrial outer membrane permeabilization (MOMP) [20]. Apoptosis-like cell death in fungi can be divided into the mitochondrial pathway, the endoplasmic reticulum pathway, and the death receptor pathway [20,21,22,23], which involves a variety of apoptosis-related proteins. Apoptosis-like PCD in fungi can be categorized into the mitochondrial pathway, the endoplasmic reticulum pathway, and the death receptor pathway, involving a variety of apoptosis-related proteins. Although the core regulatory components of fungal apoptosis are not yet fully understood, some factors involved in fungal apoptosis have been identified, such as the apoptosis-inducing factor (AIF) [24,25], metacaspase [26,27], inhibitors of apoptosis proteins (IAPs) [28,29], and glutathione [30]. A deeper understanding of fungal PCD responses may provide opportunities for utilizing fungal molecular apoptosis mechanisms to control fungal infections [12,31]. Most currently used antifungal drugs kill fungi through necrosis, which can often induce drug resistance in pathogens [32]. However, controlling pathogens by initiating the cell’s own PCD could avoid the emergence of drug resistance, offering another potential avenue for controlling fungal diseases [9]. Recent studies have found that epoxiconazole and metconazole can activate type I (apoptotic) and type II (autophagic) PCD. PCD in *Zymoseptoria tritici* and the combined activation of these two cell death pathways support the fungicidal activity of azole drugs in plant pathogens [8]. However, there have been no breakthrough discoveries in the targeting of PCD by natural drugs. This article mainly focuses on apoptosis-related proteins in fungi, aiming to provide a scientific basis for the development of natural agents targeting apoptosis-like PCD in fungi.

## 2. Apoptosis-Inducing Factor

The AIF is a conserved flavoprotein across the eukaryotic kingdom [24,33]. It is a caspase-independent apoptotic effector located in the mitochondrial intermembrane space [34]. The AIF mitochondrion-associated inducer of death (AMID) is a cytoplasmic homolog of AIF that functions as a mitochondrial-associated death inducer, regulating apoptosis-like PCD in a manner similar to AIF. Both AIF and AMID have a flavin adenine dinucleotide (FAD) binding motif at their N-terminus, as well as a redox enzyme domain, the nature of which is an NAD(P)H oxidase. Under normal conditions, they participate in the assembly of the electron transport chain complex I, which is essential for maintaining the optimal function of the mitochondrial respiratory chain. They play a crucial role in oxidative phosphorylation, redox control, and stress resistance [34,35]. Reactive oxygen species (ROS) are primarily generated at complexes I and III of the mitochondrial respiratory chain, especially during mitochondrial dysfunction [36]. AIF can produce superoxide radicals and regulate ROS levels [37,38]. The enhancement of ROS is a prerequisite for AIF carboxylation, proteolytic cleavage, and release from the mitochondria [39,40]. The main biochemical defect resulting from the overall reduction or specific time ablation of AIF is a defect in the respiratory chain complex I [41].

Many fungal homologs of AIF or AMID have been identified [24]. In *Saccharomyces cerevisiae*, the AIF1p (378 amino acids) has an anchoring proteolytic VRL|TV cleavage motif at the 61st amino acid site that is anchored in the mitochondria. Under apoptotic stimuli, this motif undergoes proteolysis, allowing it to translocate to the nucleus through its C-terminal nuclear localization signal, resulting in extensive chromatin condensation and DNA fragmentation [42], which are hallmarks of caspase-independent apoptosis [43]. In an *S. cerevisiae* mutant with AIF (Ynr074cp) knocked out, apoptosis-like PCD induced by H_2_O_2_ and acetic acid is significantly reduced [42]. *S. cerevisiae* lacks complex I and has three NADH dehydrogenases: NDE1, NDE2, and NDI. Among them, NDI1 is a homolog of AMID, and its overexpression in yeast leads to apoptosis-like cell death. The apoptotic effect of NDI1 overexpression is associated with increased mitochondrial ROS production [44,45]. The apoptosis-like PCD in *Cryptococcus neoformans* is also dependent on the induction of AIF1, and its deletion leads to chromosomal aneuploidy and fluconazole resistance [46]. In *Candida albicans*, the absence of AIF or AMID results in reduced ROS production under low levels of oxidative stress and weakened apoptosis-like PCD; however, exposure to high concentrations of H_2_O_2_ leads to increased ROS levels and enhanced apoptosis-like PCD [47].

Filamentous fungi typically possess several AIF or AMID homologs. In fungi such as *Podospora anserina* [25], *Neurospora crassa* [48], and *Aspergillus nidulans* [44,49], homologs of AIF or AMID play a role in apoptosis-like PCD during stress. Anacardic acid induces caspase-independent apoptosis in *Magnaporthe oryzae* by activating AIF, whose expression is upregulated in this process, suggesting that it may mediate mitochondria-dependent apoptosis through the activation of AIF [50]. In *Coprinopsis cinerea*, the AIF homolog CcAIF1 is involved in apoptosis-like PCD in the antagonistic interactions of multicellular fungi, potentially affecting the mitochondrial respiratory complex and acting as a ROS homeostasis regulator. Moreover, the overexpression of CcAIF1, combined with appropriate oxidative stress stimuli, can enhance the production of laccase in *C. cinerea* [51].

However, *A. nidulans* AifA knockout mutants exhibit reduced sensitivity to certain apoptotic stimuli but are sensitive to the oxidative stressor farnesol. Unlike mammals and other fungi, AifA in *A. nidulans* does not translocate to the nucleus under oxidative stress but is released from the mitochondria into the cytoplasm [44]. In *N. crassa*, AIF-like protein-encoding gene deletion mutants show greater resistance to the apoptotic inducers phytosphingosine and H_2_O_2_ compared to the wild-type strain [52]. In the presence of farnesol, the complex I mutant of *N. crassa* exhibits resistance to farnesol, which is associated with lower ROS accumulation [53]. There are differences between *A. nidulans* and *N. crassa* in terms of AIF-related PCD and mitochondrial function. Similarly, there are significant phenotypic differences in the overexpression of NDI1 in *A. nidulans* and *S. cerevisiae*. Overexpression of NDI1 in *A. nidulans* does not alter ROS concentrations. It increases survival rates in the presence of farnesol, while overexpression of NDI1 in *S. cerevisiae* is associated with increased mitochondrial ROS production, leading to apoptosis-like cell death. Therefore, AIF or AMID may not be a universal cell death effector, and their functions may depend on the cell type, the nature of the apoptotic damage, and their inherent DNA-binding capacity [41,44].

The cohesion subunit Mcd1/Rad21 is a nuclear phosphoprotein that plays a role not only in the cohesion of sister chromatids during mitosis and the condensation of chromosomes but also in apoptosis. In yeast, Mcd1 is cleaved during H_2_O_2_-induced apoptosis. The truncated C-terminal fragment is further processed by the ubiquitin-proteasome pathway into smaller fragments, which then translocate from the nucleus to the mitochondria (not the cytoplasm). This translocation leads to a decrease in the mitochondrial membrane potential (MMP) and cytochrome c-dependent cell death [27]. Esp1, which possesses caspase-1-like protease activity, is responsible for the cleavage of Mcd1 during H_2_O_2_-induced apoptosis in yeast [27,54]. Mcd1 is not the only substrate for Esp1; it can also cleave kinetochore/spindle proteins. The yeast homolog of Mcd1, human hRad21, is also a nuclear target for caspase-3 and caspase-7. The cleaved C-terminal products translocate from the nucleus to the cytoplasm and serve as a nuclear signal for apoptosis.

## 3. Metacaspase

In animals, the primary paradigm of apoptosis is caspase-dependent. Caspases have an extensive range of targets and are a group of apoptotic proteases that target hundreds of proteins, earning them the moniker “executioner proteases” [55]. Generally, the occurrence of apoptosis requires the activation of one or more members of the caspase family [29]. Caspases can be activated through either the intrinsic or extrinsic pathways of apoptosis, such as DNA damage, cytotoxic injury, and the activation of death receptors [56]. Fungi do not have caspases but contain metacaspases (Table 1). Metacaspases are also widely present in plants and protists [57]. In plants, metacaspases work in concert with autophagy to regulate senescence, immune responses, cell terminal differentiation, and the clearance of dead cells [58,59]. However, their function in fungi is not yet deeply understood.

### 3.1. Structural and Functional Features of the C14 Family Proteinases

**Figure 1:**
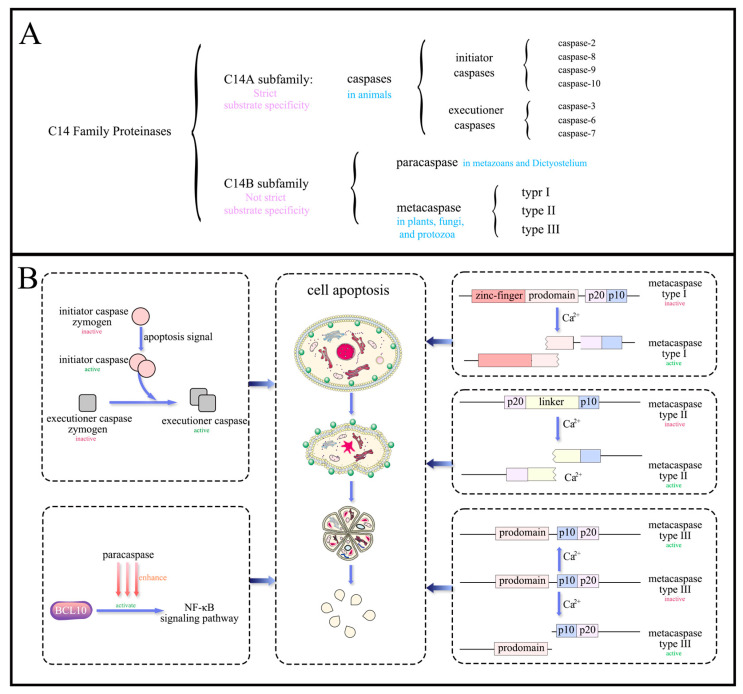
Classification of C14 family proteases (**A**) and their mechanism of inducing cell apoptosis (**B**). C14 family proteases can promote cell apoptosis in different forms, and apoptotic cells often exhibit phosphatidylserine eversion, cell wrinkling, chromatin condensation, DNA fragmentation, and the formation of apoptotic bodies.

Caspases, metacaspases, and paracaspases are all members of the cysteine-dependent (CD) clan of caspase-like cysteine proteases within the C14 family (Figure 1A) [68]. They all contain a typical peptidase C14 domain (pfam00656), also known as CASc, which features a highly conserved and distinctive α/β-fold tertiary structure known as the caspase–hemoglobinase fold (CHF). The C14 family is further divided into the C14A and C14B subfamilies. Members of the C14A subfamily are all caspases, which exhibit strict substrate specificity, cleaving peptide chains after an acidic Asp residue. The C14B subfamily includes paracaspases (which cleave after a basic Arg residue) and metacaspases (which cleave after a basic Arg or Lys residue) [69], and they have less stringent substrate specificity. From an evolutionary perspective, metacaspases and paracaspases are considered the ancestors of caspases. They differ in structural topology, substrate specificity, and activation mechanisms [57,70], with metacaspases showing a broader structural variation compared to caspases and paracaspases [71].

The C14 family of proteases is expressed as zymogens and undergo structural modifications before activation. Caspases and paracaspases are activated upon dimerization, while metacaspases do not undergo dimerization but most still require autoprocessing before activation (Figure 1B). The autoprocessing activity of the vast majority of metacaspases is highly dependent on Ca^2+^, influenced by the local concentration changes of Ca^2+^ ions and pH levels. Interestingly, metacaspases contain an additional N-terminal Cys, which can take over proteolytic activity after the zymogen has undergone autoprocessing. The activity of C14 proteases is also regulated by post-translational modifications, such as phosphorylation, ubiquitination, nitrosylation, and direct interactions with other proteins. Metacaspases are further categorized into three types: Type I has a proline-rich domain at the N-terminus with a zinc-finger motif [72]; Type II lacks an N-terminal domain, but its CHF p20 and p10 subunits are separated by a long linker; Type III has the gene order of the p20 and p10 subunits reversed relative to Type I and II. Caspases and some Type I metacaspases remove the N-terminal prodomain and cleave the linker between the catalytic large and small subunits to achieve self-maturity.

### 3.2. Regulation of Fungal Apoptosis by Metacaspase

Apoptosis-like cell death in fungi can be either metacaspase-dependent or metacaspase-independent, usually mediated by mitochondria [15]. The first metacaspase with cellular function identified in fungi is YCA1 (formerly known as Mca1) in *S. cerevisiae* [65]. In the presence of the normal YCA1/Mca1, when *S. cerevisiae* is exposed to H_2_O_2_, many markers of PCD appear, such as the accumulation of ROS, phosphatidylserine (PS) externalization, and nuclear condensation. The deletion mutant *Δyca1* does not undergo apoptosis upon H_2_O_2_ induction and leads to a large accumulation of oxidized proteins dependent on H_2_O_2_, upregulation of 20S proteasome activity, and inhibition of ubiquitination activity. Overexpression of *yca1*, on the other hand, increases H_2_O_2_-induced caspase-like activity and the intensity of apoptosis [73,74,75]. In *S. cerevisiae*, mutations in DNA replication initiation proteins weaken the initiation of DNA replication and checkpoints, also inducing apoptosis-like characteristics observed in metazoans, including the production of ROS and the activation of metacaspase Yca1p. In at least one initiation mutant, the activation of Yca1p occurs in the early stages of cell death. It contributes to the lethal effect of the mutation carried by the strain apoptosis in initiation mutants, which may be induced by DNA damage [76].

YCA1/Mca1 possesses a protein quality control (PQC) function, participating in the proteasome-dependent degradation of misfolded or unfolded proteins, which contributes to cellular homeostasis and extends the lifespan of cells [26,77]. Whether YCA1/Mca1 is an angel or a demon, whether it initiates PCD or extends the lifespan, may depend on the situation and be regulated through metacaspase-dependent or -independent mechanisms for self-homeostasis of these proteins [78]. It may be closely related to the threshold concentration of YCA1/Mca1 within the cell.

In *M. oryzae*, two metacaspases homologous to the yeast YCA1/Mca1, MoMca1, and MoMca2 have been identified. When overexpressed in yeast, they induce a reactive oxygen species-dependent cell death (RCD) response. Mutants of *M. oryzae* lacking MoMca1 and MoMca2 exhibit higher growth rates under oxidative stress and lead to the accumulation of insoluble protein aggregates [60]. In *P. anserina*, RCD in aging cultures is induced by oxidative stress and occurs after metacaspase activation [25,63]. The deletion of the metacaspases PaMCA1 and PaMCA2 extends the lifespan of the aging culture; thus, in contrast to the function of YCA1/Mca1 in yeast, PaMCA1, and PaMCA2 are considered to be catalysts of aging in *P. anserina* [19]. When *Penicillium chrysogenum* is treated with S-ethyl ethanethiosulfinate (ALE), the spores undergo morphological changes associated with apoptosis, including loss of plasma membrane integrity and exposure to PS. After treatment of mycelium with ALE, ROS-dependent metacaspase activity can be detected [79]. Mutants of *N. crassa* with metacaspase gene deletions or AIF gene deletions are unaffected by heterokaryon incompatibility (HI)-mediated PCD/RCD, proving that HI-mediated PCD/RCD requires metacaspase or AIF [80,81]. The transcription factor vib-1, a member of the NDT80 (a p53-like) superfamily, is necessary for HI-mediated cell death in the *N. crassa* and shows genetic interaction with a kinase (IME-2) [11,62].

Despite *Aspergillus* species possessing many downstream components of the mammalian apoptotic pathway, including several proteins not found in *S. cerevisiae* [81], there appears to be no association between caspase-like activity and metacaspases in *Aspergillus fumigatus* and *A. nidulans*. It has been demonstrated that there is strong caspase-like activity against specific substrates of caspase-1 and caspase-8 within *Aspergillus* cells, and the apoptotic phenotype can be blocked by the specific caspase inhibitor Z-FAD-fmk [82]. However, the deletion of genes encoding metacaspases (*casA* and *casB*) has little effect on measurable caspase activity, mycelial viability, or pathogenicity and may even be protective. This suggests that the caspase activity is independent of metacaspases [63,64,75,83]. In contrast, *P. digitatum* encodes two metacaspases, CasA and CasB, and its self-produced AfpB can induce cell RCD, increasing the expression of metacaspase, *fadA* (G protein α-subunit), *nma* (homolog of the human HtrA2 pro-apoptotic gene), *aif1*, and *amid2* in the mycelium [61]. Therefore, understanding whether metacaspases are involved in apoptosis-like cell death in fungi and their molecular mechanisms is still largely elusive [14], with no consensus. Perhaps the key protein factors involved in apoptosis vary greatly among different fungi. Given that there are currently no specific molecular probes for measuring and inhibiting metacaspase activity, the potential role of metacaspases in fungal apoptosis and lifespan extension awaits further evidence for elucidation. The genome of the slime mold *Dictyostelium discoideum* only encodes a single paracaspase, PCP, but its PCD seems to be independent of PCP activity [84].

The elucidation of the role of metacaspases in the signaling pathways that control the life and death, development, and aging of fungi is crucial for a correct understanding of fungal PCD or RCD and for establishing a paradigmatic program for fungal apoptosis. Recent studies suggest that the caspase-centric view of death should be re-evaluated. Apoptosis may merely represent an extreme form of caspase-mediated cellular transformation. Under conditions of acute endoplasmic reticulum (ER) stress, the trimming of the nuclear pore complex by caspases may be a stress-compensatory mechanism to suppress mRNA output and reduce the load on protein synthesis and folding. In chronic ER stress scenarios, where caspases persist over an extended period, this could potentially jeopardize cellular homeostasis [55]. Therefore, there is reason to question whether metacaspases in fungi are centered on death.

## 4. Inhibitors of Apoptosis Proteins

The IAPs are renowned for their anti-apoptotic activity and their ability to directly bind and inhibit caspases [85]. In living cells at a steady state, IAPs promote the degradation of active caspases and AIF through ubiquitination. IAPs were first discovered in baculoviruses of insects and are widely present in eukaryotes [14,28,86,87], being highly conserved at the level of sequence and function. In mammals, IAPs are effective regulators of caspase activity. They can modulate innate immune responses by inhibiting caspase-dependent and -independent cell death [88,89], as well as regulating autophagy and cell division [90]. IAPs are also key to cancer cells evading apoptosis, making them a critical target for cancer therapy [91,92]. Recent studies suggest that IAPs also have functions beyond inhibiting cell death. Although IAPs in mammalian, insect, and viral systems have been extensively studied, the mechanisms of action of fungal IAPs remain largely unknown. The mammalian IAP family comprises eight members: XIAP, cIAP1, cIAP2, ML-IAP, NAIP, ILP2, Survivin, and Bruce [85,88]. However, the members of the fungal IAP family are still not well understood.

### 4.1. Structural Characteristics of Inhibitors of Apoptosis Proteins

**Figure 2:**
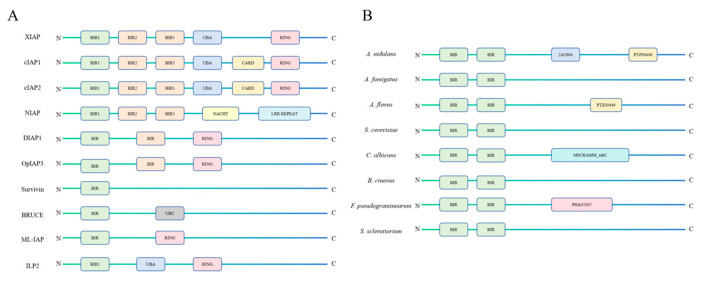
The structure of different types of inhibitors of apoptosis proteins (IAPs) [85]. Different types of IAPs and their structures in (**A**) animal cells and (**B**) fungal cells. The N-terminus of IAPs contains a specific BIR domain, while the C-terminus structure varies among different species, such as the UBA domain and RING domain in animal cells IAPs and the PTZ00449 domain in fungal cells IAPs.

The characteristic structural feature of IAPs is the conserved and unique Baculovirus IAP Repeat (BIR) domain at their N-terminus. The BIR domain, approximately 70 amino acid residues in length, is a zinc-binding domain that facilitates protein–protein interactions, stabilized by the coordination of a Zn^2+^ ion with three conserved Cys and one His residue to maintain the BIR fold [85,90]. BIRs can be divided into two types, Type I and Type II, based on the presence or absence of a deep peptide-binding groove. Type II BIRs contain a unique hydrophobic cleft that binds to specific N-terminal IAP-binding motifs (IBM) of interaction partners such as caspases or IAP antagonists [85]. Type I BIRs possess only a shallow pocket and cannot interact with caspases or IAP antagonistic proteins, targeting other functional proteins instead. Some IAPs coexist with both Type I and Type II BIRs, ensuring that they can not only regulate apoptosis but also participate in other cellular processes. In addition to the BIR domain, some animal IAPs have a carboxy-terminal Really Interesting New Gene (RING) zinc finger domain or a zinc-independent U-box domain that is similar to RING (Figure 2A). This domain allows IAPs to function as E3-ligases, responsible for the ubiquitination and degradation of caspase-related proteins and themselves, thereby inhibiting caspase activity [85,93,94]. However, although fungal IAPs are, on average, several hundred amino acids larger than those of mammals, insects, or viruses, they lack the RING domain. They are unlikely to undergo auto-ubiquitination regulation, suggesting that there may be a regulatory mechanism in fungi that differs from other eukaryotes [93]. IAPs also contain a ubiquitin-associated domain (UBA) and a ubiquitin-conjugating domain (UBC), allowing them to bind to poly-ubiquitinated proteins and substrates [85]. Some IAPs also have a caspase activation and recruitment domain (CARD). A typical IAP usually contains two types of BIR domains (Type I and Type II), a ubiquitin-associated (UBA) domain, and a RING domain (Figure 2A).

Fungal IAP-like proteins also possess a specific BIR domain, commonly referred to as the Bir1 protein. In fact, IAPs from different fungal species are highly conserved, all containing two BIR domains, but the C-terminal domains vary greatly among different species. This suggests that fungal IAPs may regulate similar processes through their BIR domains and have evolved to regulate distinctly different species-specific processes through their variable C-terminal domains (Figure 2B) [2,94,95,96]. AnBir1 is an IAP from *A. nidulans*, predicted to have 2A1904 and PTZ00449 superfamily domains at its C-terminus. CaBir1 is an IAP from *C. albicans*, predicted to have an MSCRAMM_SdrC superfamily domain, which includes a YSIRK-type signal peptide and an LPXTG protein anchoring motif. FpBir1 is an IAP from *Fusarium pseudograminearum*, which has a PHA03307 superfamily domain. Proteins of this family have ICP4-type transcription factor functions: ICP4 is a viral nucleoprotein that binds double-stranded DNA and may target silent histones [97,98].

### 4.2. The Function of Fungal Inhibitors of Apoptosis Proteins

IAPs in different fungi often have different effects on apoptosis, growth, development, and pathogenicity (Table 2). In *S. cerevisiae*, IAPs are localized to both the nucleus and the cytoplasm, playing a crucial role in regulating various biological processes occurring in these compartments, including the cell cycle and cell death [14]. In *Cryphonectria parasitica*, the causative agent of chestnut blight, CpBir1, is preferentially localized to the nucleus in young cultures, but in aged cultures, it is localized to both the nucleus and the cytoplasm [99,100]. The shuttling between the nucleus and the cytoplasm is essential for the myriad processes known to be regulated by IAPs.

In *S. cerevisiae* and *Schizosaccharomyces pombe*, phosphorylated Bir1 (Bir1p) forms the kinetochore passenger complex (KPC) with multiple proteins [27], which is essential for chromosome condensation and the fidelity of chromosome segregation. The functions of IAPs in different fungi are shown in Table 2. Typically, the deletion of IAPs in fungi leads to chromosomal mis-segregation, chromosomal loss (which can be rescued by overexpression of the C-terminus of BIR1), and typical apoptotic phenotypes. Overexpression of IAPs significantly reduces metacaspase activity and delays cell death [95]. Additionally, the deletion mutant of BIR1 in *S. cerevisiae* exhibits no phenotypic changes when grown in a rich medium. Still, it shows reduced efficiency in spore formation and pseudohyphal differentiation under nutrient starvation [86,99]. The gene encoding the IAP protein MoBir1 in *M. oryzae*, when heterologously expressed in yeast, inhibits H_2_O_2_-induced cell death and delays the chronological aging of yeast cells. Delayed aging was found to require the carboxy-terminal of MoBir1 [2,94]. In *A. fumigatus* and *A. nidulans*, growth can only be observed when BIR1 is induced to express [82].

Fungal IAPs are involved in host-pathogen interactions. Pulmonary neutrophils and macrophages engulf the conidia of *A. fumigatus* and induce PCD mediated by oxidative stress [82]. Spores engulfed by macrophages and neutrophils exhibit reduced viability, increased DNA fragmentation, and enhanced induction of caspase-like activity, indicating that host-induced PCD is an important mechanism for clearing *A. fumigatus* infections [82]. In the early stages of the interaction between *Botrytis cinerea* and host plants, the host induces a large amount of cell death in germinating spores, characterized by chromatin condensation and DNA breaks. However, *B. cinerea* slowly recovers from the host-induced attack and establishes infection, a process mediated by BcBir1 [101]. Typically, the deletion of fungal IAPs leads to reduced virulence and infectivity, while overexpression of IAPs results in higher virulence or pathogenicity. However, the performance in *M. oryzae* is unusual; the overexpression mutant of MoBIR exhibits reduced virulence, which is related to the impaired ability of the strain to accumulate ROS [2,94].

## 5. Inhibitors of Apoptosis Proteins Antagonists

In apoptotic animal cells, IAP antagonists inactivate IAPs, leading to the derepression of active caspases [88]. Mammalian IAP antagonists include the mitochondrial protein Smac (which is released from the mitochondria into the cytoplasm during the induction of apoptosis), Omi/HtrA2, XIAP-associated factor 1 (XAF1), and ARTS. IAPs can also slow down apoptosis by degrading Smac and ARTS. Smac and Omi/HtrA2 contain a conserved four-amino acid motif (AVPI/F), known as the IAP-binding motif (IBM). SMAC interacts with the BIR domain of IAPs through the IBM, thereby antagonizing IAPs [103]. IAP antagonists are localized in the mitochondrial intermembrane space in some organisms and in the cytoplasm in others [103,104]. Different IAP antagonists have distinct mechanisms of action; some compete for binding to caspases and promote auto-ubiquitination to inhibit activity [20], while others inhibit IAP activity by binding and irreversibly cleaving IAPs.

In fungi, a major class of IAP antagonists consists of homologs of the serine protease HtrA2/Omi, which have been confirmed in *S. cerevisiae* and *B. cinerea* and are the main negative regulators of fungal IAPs [15,17,99]. The first HtrA2/Omi homolog identified in yeast was Nma111p, and subsequently, the IAP negative regulator BcNma in *B. cinerea* was identified through homology with Nma11p [105,106]. Both Nma111p and BcNma possess pro-apoptotic functions. Under conditions that induce PCD, the levels of chromatin condensation and DNA fragmentation are reduced in *Δnma111* and *Δbcnma* mutants, with an increased survival rate. In contrast, overexpression of these genes results in the opposite phenotype. When *bcnma* is expressed in *Δnma111* cells, the sensitivity of the cells to oxidative and temperature stress conditions is restored to wild-type levels, indicating that BcNma functionally complements Nma111p, and their functions are conserved [105,106].

Nma111p and BcNma are localized exclusively to the nucleus [99,105]. In yeast, Nma111p interacts with Bir1p through its N-terminal domain and utilizes its serine protease activity to proteolytically cleave Bir1p. This degradation is associated with an increase in apoptosis-like cell death [99]. Bir1p is localized in both the nucleus and the cytoplasm, but Nma111p is found only in the nucleus, suggesting that Bir1p in the cytoplasm may be regulated by other proteins.

## 6. Cytochrome C

Cytochrome c (cyt-c) plays a crucial role in respiration and apoptosis. Its translocation from the mitochondria to the cytoplasm is a key event in apoptosis, directly associated with a decrease in cyt-c oxidase activity [20]. Mitochondrial depolarization leads to the release of cyt-c and other pro-apoptotic factors into the cytoplasm, resulting in the activation of the yeast caspase-like protease Mca1, which in turn activates a cascade of apoptosis. This form of cell death is most common in yeast. The release of cyt-c can halt electron transfer, leading to the loss of MMP and an increase in the production of ROS. Novel pyrrole-based 1,2,3-triazole derivatives arrest the cell cycle of *Candida auris* in the S phase and induce apoptosis, causing cyt-c to move from the mitochondria to the cytoplasm accompanied by membrane depolarization [107].

## 7. Glutathione

Glutathione exists in two forms: the reduced form (GSH) and the oxidized form (GSSG), with glutathione reductase catalyzing the interconversion between these two states. Under physiological conditions within cells, GSH predominates and plays a critical role in combating oxidative stress [108], being consumed in reactions that eliminate harmful compounds to protect the cell. Apoptosis is associated with glutathione depletion. Under apoptotic stimuli, glutathione is expelled from the cell, altering its reducing power and leading to oxidative stress, which triggers apoptosis. An ATP-dependent process mediated by plasma membrane glycoproteins facilitates the release of GSSG. The GSH/GSSG ratio is typically tightly regulated to maintain cellular homeostasis, and perturbations in this ratio can affect the redox state and induce apoptosis. Null mutants lacking glutathione synthesis exhibit increased ROS and undergo apoptosis, which can be rescued by glutathione supplementation. In *C. albicans*, the ABC transporter genes *CDR1* and *CDR2* (involved in drug resistance) encode ATP-dependent plasma membrane efflux pumps that participate in cellular detoxification, stress response, and drug resistance. Farnesol, when bound to intracellular glutathione, disrupts the intracellular redox balance, leading to apoptosis.

Farnesol-induced apoptosis in *C. albicans* is mediated through the efflux by CDR1-p and the depletion of intracellular glutathione [30]. It is known that lipophilic compounds such as farnesol conjugate with glutathione, leading to a significant decrease in intracellular glutathione levels, while the upregulation of CDR1 and a decrease in cell viability occur concurrently. In mutant strains lacking CDR1, the toxicity of farnesol is reduced, while in strains overexpressing CDR1, its toxicity is increased. In aged cultures, the secretion of farnesol by fungi increases, suggesting that *C. albicans* cells may have evolved a PCD mechanism with evolutionary advantages by regulating the production of farnesol. Farnesol concentration and exposure time-dependently reduce the total glutathione levels while decreasing the viability of *C. albicans*. Exogenous glutathione supplementation not only maintains intracellular glutathione levels and increases survival rates but also significantly reduces ROS accumulation and caspase activation. The GSH content is directly proportional to the concentration of farnesol, with the lowest intracellular content in strains overexpressing CDR1 and the highest in strains lacking CDR1. Farnesol can trigger apoptosis-like characteristics in *Rhizoctonia solani* AG1-IA, such as the production of ROS and an increase in superoxide dismutase (SOD) activity, which may lead to the simultaneous occurrence of necrosis and apoptosis [109].

At low cadmium concentrations, *S. cerevisiae* undergoes glucose-dependent PCD, the apoptotic features of which are eliminated in a metacaspase Yca1p knockout mutant, indicating that it is dependent on metacaspase Yca1p. Cadmium-dependent apoptosis is also inhibited in the *gsh1* mutant, demonstrating that the PCD process requires GSH [109]. This suggests that both the caspase pathway and the cellular redox balance maintained by GSH play crucial roles in mediating cadmium-induced PCD in yeast [110].

## 8. Other Apoptosis Related Proteins

In *S. cerevisiae*, the *UBP10* gene encodes a deubiquitinating enzyme that is significant for resisting apoptosis, and its inactivation can lead to the appearance of typical apoptotic characteristics. Research by Bettiga et al. has shown that yeast metacaspase is involved in the active cell death triggered by the loss of UBP10 function [111]. The Ca^2+^/calmodulin/calcineurin signaling pathway may influence the death response in fungi [31]. Poly(ADP-ribose) polymerase, a classic substrate of caspase-3 during apoptosis, has been demonstrated to undergo transferase-dependent proteolysis in the process of fungal PCD [112]. Glyceraldehyde-3-phosphate dehydrogenase (GAPDH), a substrate of caspase-1 during infection or septic shock, is cleaved by YCA1 in a metacaspase-independent manner during H_2_O_2_-induced yeast cell death [113]. Nucleotide-binding leucine-rich repeat proteins (NLR) are a large class of intracellular immune receptors found in animals, plants, and fungi and are involved in microbial defense. In *Arabidopsis thaliana*, the metacaspase AtMC9 cleaves the NLR-dependent immune negative regulator AtCDC48A [114]. Autophagy and protein kinase C are involved in the farnesol-induced apoptosis [44], and interestingly, the fungal homolog of CDC48 is predicted to be a substrate of metacaspase during the farnesol-induced cell death [115]. In yeast, components related to cell cycle checkpoints, such as cyclin-dependent kinases (CDKs), have also been shown to participate in apoptotic responses. This helps to strictly control the cell death of cells with defects in cell cycle regulation [27]. The pro-apoptotic members of the BCL-2 protein family, Bax and Bak, have been shown to kill yeast cells when heterologously expressed in yeast [116,117]. Not all fungi respond in the same way to heterologous pro-apoptotic proteins; the expression of Bax in *Pichia pastoris* leads to growth arrest, accompanied by chromatin condensation and the accumulation of autophagic bodies, but without other apoptotic characteristics [118]. A targeted proteomics study on 32 apoptosis-related proteins in yeast has revealed that the oxidoreductase Oye32 may be a common apoptotic marker for a variety of stressors [119].

## 9. Summary and Prospect

**Figure 3:**
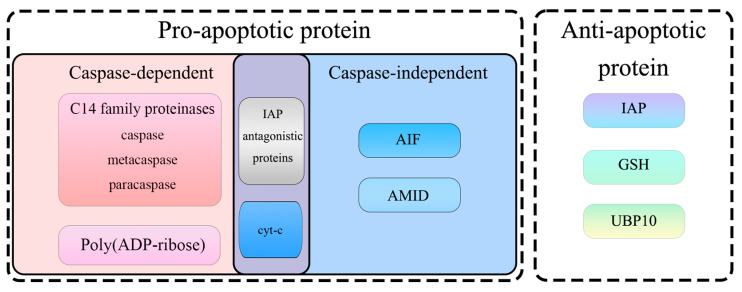
Classification of apoptosis-related proteins.

In summary, fungal apoptosis-related proteins can be mainly divided into pro-apoptotic proteins and anti-apoptotic proteins (Figure 3). AIF, metacaspase, and IAP antagonists, along with cyt-c, are considered pro-apoptotic proteins; IAPs and glutathione are anti-apoptotic proteins. The specific functions of these proteins are summarized as follows: (1) AIF, which can produce superoxide radicals, regulate ROS levels, and induce apoptosis. Enhanced ROS is a prerequisite for AIF carboxylation, proteolytic cleavage, and release from mitochondria. (2) Metacaspase, widely present in plants, fungi, and protozoa. The expression level of metacaspase can positively regulate the intensity of hydrogen peroxide-induced apoptosis and has PQC functions. (3) IAPs, which can directly bind to and inhibit caspases, promoting the degradation of active caspases and AIF through ubiquitination. IAPs can regulate cell cycle and cell death and are involved in host-pathogen interactions. (4) IAP antagonists, which can inactivate IAPs, remove the inhibitory effect of IAPs on caspases, and promote cell apoptosis. (5) Cyt-c. As an important part of the electron transport chain, the release of cyt-c can prevent electron transfer, reduce MMP, and promote ROS production. (6) Glutathione. The ratio of GSH/GSSG is crucial for maintaining cellular homeostasis. Disturbance of this ratio can affect the redox state and induce cell apoptosis.

**Figure 4:**
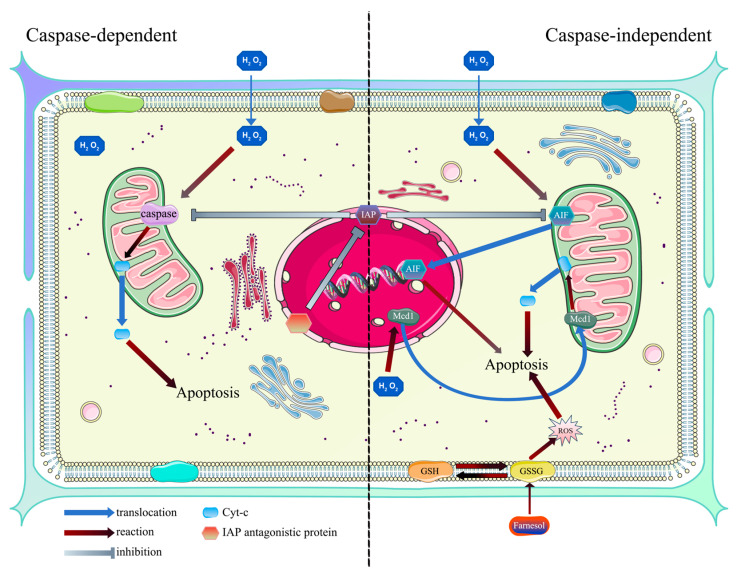
Basic model of fungal apoptosis. The blue arrows represent translation, and the deep red arrows represent reaction.

Fungal apoptosis can be categorized into two types: caspase-dependent and caspase-independent (Figure 4). Metacaspase and IAP antagonists are involved in caspase-dependent apoptosis, while AIF and glutathione are involved in caspase-independent apoptosis. IAPs and cyt-c play significant roles in both types of apoptosis. In caspase-dependent apoptosis, under the action of caspases located in the mitochondrial intermembrane space, cyt-c is released into the cytoplasm, leading to apoptosis. IAPs can suppress the apoptotic effect of caspases, while IAP antagonistic proteins can impede the function of IAPs. In caspase-independent apoptosis, AIF can translocate from the mitochondrial intermembrane space to the nucleus, inducing apoptosis. Of course, all of this happens when the fungus is under exogenous or endogenous stress, such as H_2_O_2_, farnesol, or other events that may produce ROS.

This is despite the fact that it has been recognized that apoptosis plays a crucial role in the growth, development, invasion, and virulence of pathogenic fungi. However, the knowledge of fungal apoptosis-like cell death is still limited and incomplete. There are still several issues to be solved: (1) At present, most research on fungal apoptosis is still at the level of visual observation, lacking in-depth analysis of key molecules and apoptotic pathways, which is not enough to construct a relatively complete apoptotic model map. (2) Because the known apoptosis-inducing factors and patterns of fungal cells are not uniform, the sequence of various apoptosis-inducing features is not clear, and no universal apoptosis-inducing “switch” or signaling pathway common to most fungi has been found. This provides a challenge to clarify the trigger factors and inhibition modes of fungal apoptosis. (3) Currently, the methods used to study fungal apoptosis mainly follow the detection methods of mammalian apoptosis, and specific methods such as molecular probes for researching fungal apoptosis are desperately lacking, which makes it difficult to explore the exact mechanism of fungal apoptosis. (4) The development of antifungal drugs that target apoptosis as their mechanism of action is lagging behind. The structure of azole drugs widely used in agricultural antifungal treatment is similar to that of azole drugs used clinically. Their mechanism of action is to inhibit fungi by suppressing cell wall integrity rather than killing fungi, leading to increased drug resistance with long-term use. Research indicates that inducing fungal apoptosis and inhibiting cell wall integrity are distinct pathways. Utilizing natural drugs that trigger endogenous fungal apoptosis for the prevention and treatment of fungal diseases can effectively avoid the development of drug resistance, offering higher target specificity, safety, and practicality. However, due to the lag in the study of fungal apoptosis, the development of targeted drugs that directly act on apoptotic proteins and pathways is severely limited.

There is still a large gap in the research on fungal apoptosis. Future research should delve deeper into the factors and mechanisms of fungal apoptosis and lay a foundation for the development of new natural drugs targeting the activation of fungal endogenous cell suicide.

## Figures and Tables

**Table 1:** Metacaspases in fungi.

Fungi	Metacaspase/Paracaspase	Reference
*Magnaporthe oryzae*	MoMca1, MoMca2	[60]
*Penicillium digitatum*	CasA, CasB	[61]
*Ustilago maydis*	Mca1	[26]
*Neurospora crassa*	IME-2	[62]
*Podospora anserina*	PaMCA1, PaMCA2	[25,63]
*Aspergillus fumigatus*	CasA, CasB	[64]
*Saccharomyces cerevisiae*	Mca1/YCA1, Esp1 (caspase-1 like protease)	[54,65]
*Schizosaccharomyces pombe*	PCA1	[66,67]

**Table 2:** IAPs in fungi and their effects on apoptotic programmed cell death (PCD), growth and development, and pathogenicity.

Fungi	IAP	Gene Knockout or Knockdown	Gene Overexpression	Reference
*Fusarium pseudograminearum*	FpBIR1	✧ Mainly regulates cell apoptosis during the conidia stage. More sensitive to ROS and higher nuclear breakage. The germination is significantly reduced. ✧ The branching of hyphae decreases, and the production of conidia is less lethal. ✧ The pathogenicity of wheat and barley leaves is significantly reduced.		[96]
*Saccharomyces pombe*	BIR1	✧ Apotosis.		[95]
*Cryphonectria parasitica*	CpBIR1	✧ There are more apoptotic cell nuclei and accelerated aging. ✧ Decreased growth rate, reduced biomass accumulation, no formation of conidia, extreme sensitivity to oxidative stress, and sharp decline in radial growth under H_2_O_2_ stress. ✧ The pathogenicity of chestnut stems has also been weakened.	✧ Enhanced resistance to oxidative stress. ✧ Delaying aging.	[96]
*Magnaporthe oryzae*	MoBIR1	✧ Accelerate aging. ✧ There is no significant phenotypic change which is possibly due to insufficient reduction in gene expression.	✧ Reduce ROS and decrease PCD phenotype. ✧ Higher growth rate, more aerial hyphae, increased biomass, decreased autolysis, enhanced resistance to H_2_O_2_, and delayed hyphal aging. ✧ The virulence has not been enhanced, and there are fewer and smaller lesions on the host leaves.	[2,94]
*Aspergillus fumigatus*	AfBIR1	✧ High sensitivity to sub apoptotic H_2_O_2_ levels, higher caspase like activity, higher DNA fragmentation, and reduced survival ability.	✧ Increased resistance to apoptotic PCD Higher survival rate, fewer DNA fragments, and lower caspase-like activity. ✧ It does not show an increase in biomass and has no effect on spore germination. ✧ Higher survival rate, higher germination rate, and higher infection rate. Higher toxicity, more severe lung tissue damage, and higher mortality rate.	[82]
*Botrytis cinerea*	BcBIR1	✧ The degree of pigmentation is higher. ✧ Partial knockout strains showed higher PCD phenotype enhancement under H_2_O_2_ stress. Abnormal branching and swelling are observed in older/long-term cultures. ✧ More sensitive to host induced PCD.	✧ Enhanced resistance to cell death induction conditions and decreased PCD phenotype. ✧ Growth and biomass accumulation have both increased. ✧ Has higher resistance and stronger virulence to PCD induced by hosts such as *Arabidopsis thaliana.*	[100,101]
*Saccharomyces cerevisiae*	BIR1	✧ Show typical apoptotic phenotype. ✧ Chromosomal segregation and loss.	✧ Delayed death leads to significant decrease in meta caspase activity.	[102]
